# Supplementary material for: The adder (Vipera berus) in Southern Altay Mountains: population characteristics, distribution, morphology and phylogenetic position
Source: PeerJ. 2016 Aug 16;4:e2342. doi: 10.7717/peerj.2342 (PMC4991858; doi:10.7717/peerj.2342)
Supplement: Table S1 [file peerj-04-2342-s001.docx]

| **Taxon** | **Sample Code** | **Location** | **Accession number** |
| --- | --- | --- | --- |
| *V. b. berus* | SW_ Switzerland031 | Vallée de Joux Switzerland | DQ186031 |
|  | SW_ Switzerland032 | Uri, Switzerland | DQ186032 |
|  | SW_ Switzerland033 | Préalpes fribourgoises, Switzerland | DQ186033 |
|  | NW_Sweden041 | Mora, Sweden | DQ186041 |
|  | NW_Sweden043 | Långalma, Sweden | DQ186043 |
|  | NW_Sweden046 | Grebbestad, Sweden | DQ186046 |
|  | E_Russia050 | Shulgino, St Petersburg Prov, Russia | DQ186050 |
|  | E_Russia051 | Dedilovskie Vysetki, Tula Prov., Russia | DQ186051 |
|  | E_Russia052 | Altai, Russia | DQ186052 |
|  | E_Russia053 | Krasnoyarski Kray, Russia | DQ186053 |
|  | E_Poland059 | Ustrzyki Dolne, Poland | DQ186059 |
|  | SW_Italy066 | Ugorizza, Italy | DQ186066 |
|  | SW_Switzerland067 | Pontresina, Switzerland | DQ186067 |
|  | SW_Switzerland068 | Pontresina, Switzerland | DQ186068 |
|  | SW_Italy069 | Pontebba, Italy | DQ186069 |
|  | SW_Italy070 | Val Cimoliana, Italy | DQ186070 |
|  | E_Russia730 | Perm, Russia | KC176730 |
|  | SW_Switzerland105 | Vaud, Switzerland | FR727105 |
|  | SW_Switzerland104 | Graubünden, Switzerland | FR727104 |
|  | Southern Altay | Southern Altay Mountains, China | KU942378* |
| *V. b. sachalinensis* | Russian Far East054 | Yuzhno-Sakhalinsk, Russia | DQ186054 |
|  | Russian Far East055 | Khabarovsky Kroy, Russia | DQ186055 |
| *V. b. bosniensis* | Balkan_Montenegro073 | Bjelasica mountain, Montenegro | DQ186073 |
|  | Balkan_Serbia074 | Kopaonik mountain, Serbia | DQ186074 |
|  | Balkan_Serbia075 | Stara Planina mt., Serbia | DQ186075 |
|  | Balkan_Bosnia076 | Treskavica, Bosnia | DQ186076 |
|  | Balkan_Bosnia077 | Bihac, Bosnia | DQ186077 |
|  | Balkan_Bosnia078 | Slavonski Brod, Bosnia | DQ186078 |
|  | Balkan_Bulgaria079 | Musala Mountain, Bulgaria | DQ186079 |
|  | Balkan_Bulgaria080 | Pirin Mountain, Bulgaria | DQ186080 |
| *Vipera seoanei* | *V. seoanei* | Spain | DQ186030 |
| *Vipera altaica* | *V. altaica* | Kazakhstan | KC176729 |
| *Vipera renardi* | *V. renardi* | Krym, Ukraine | KC316108 |
| *Vipera ursinii* | *V. ursinii* | Turkey | KC316113 |

* The other eight samples' sequences with respective specimen voucher were submitted to GenBank with accession numbers KX345249 to KX345256.
